# Promotion of HIV clearance by sensitization of HIV reservoirs to cell death

**DOI:** 10.3389/fimmu.2025.1600741

**Published:** 2025-07-14

**Authors:** Min Li, Baichao Sun, Jing Dong, Jian-Rong Li, Laurie J. Minze, Min Chen, Chao Cheng, Jin Wang

**Affiliations:** ^1^ Immunobiology and Transplant Science Center, Houston Methodist Research Institute, Houston, TX, United States; ^2^ Institute for Clinical and Translational Research, Baylor College of Medicine, Houston, TX, United States; ^3^ Department of Pathology and Immunology, Baylor College of Medicine, Houston, TX, United States; ^4^ Department of Medicine, Baylor College of Medicine, Houston, TX, United States; ^5^ Dan L Duncan Comprehensive Cancer Center, Baylor College of Medicine, Houston, TX, United States; ^6^ Department of Surgery, Weill Cornell Medical College, Cornell University, New York, NY, United States

**Keywords:** HIV, T cell reservoirs, apoptosis, single cell RNA sequencing, cell death

## Abstract

**Introduction:**

HIV integrates its proviral DNA into the host genome to establish persistent infection. To promote HIV clearance, we have designed an approach for selective elimination of host cells harboring replication-competent HIV (SECH), through inhibition of autophagy and anti-apoptotic molecules during viral reactivation. SECH approach can clear HIV-infected cells in approximately 50% humanized mice. However, the mechanisms for the resistance of reservoirs to depletion in mice with failure in HIV clearance are unclear.

**Methods:**

We have performed single cell transcriptome analyses of HIV-infected T cells that escaped the treatments, in order to identify cellular pathways that could be targeted to facilitate the deletion of refractory HIV reservoirs.

**Results:**

By single cell RNA sequencing analyses of T cell reservoirs resistant to SECH treatments, we found increases in pro-survival autophagy and glycolysis. Moreover, these resistant reservoirs expressed more epigenetic modifiers that repress HIV gene expression, while targeting such epigenetic repression promoted cell death in HIV-infected cells.

**Discussion:**

Our results indicate that T cell reservoirs refractory to depletion maintain a delicate balance between low levels of HIV gene expression and evasion of cell death. This study suggests that targeting epigenetic repression of HIV is critical for the depletion of the viral reservoirs.

## Introduction

HIV undergoes reverse transcription and integration of its proviral DNA into host genome to establishes persistent infections. While the expression of cytopathic HIV gene products can cause apoptosis in a portion of infected cells, the surviving cells that establish latent infections can persist and represent a major obstacle to an HIV cure. Current antiretroviral therapy (ART) can effectively inhibit active viral replication, and prevent the onset of the clinical symptoms of acquired immunodeficiency syndrome. However, continuous ART is necessary to prevent HIV rebound from latent HIV reservoirs (1–4). Strategies such as immunotherapy, HIV vaccines, development of neutralization antibodies, excising the proviral DNA by CRISPR-Cas9, and “lock-and-block” to epigenetically repress HIV, have been tested for the control of HIV reservoirs (5–15). However, a cure method to delete the reservoirs containing the intact HIV proviruses capable of producing new infectious viruses remain to be developed.

The molecular mechanisms why HIV reservoirs in people living with HIV (PLWH) are refractory to depletion remain poorly understood. The “shock-and-kill” strategy employs latency reversal agents (LRAs) to induce the expression of cytopathic HIV genes to kill host cells. However, using LRAs alone is not effective in reducing HIV reservoirs in patients (16), suggesting that HIV-infected cells cannot be killed efficiently by this approach *in vivo*. Indeed, we have found that stimulation of HIV-infected T cells can induce the expression of pro-survival autophagy and anti-apoptotic molecules (17). These pro-survival mechanisms likely counteract cell death induced by HIV genes, leading to inefficient deletion of HIV reservoirs (17).

We have therefore developed an approach for selective elimination of host cells harboring replication-competent HIV (SECH), through inhibition of autophagy and anti-apoptotic molecules during HIV re-activation (17–19). The SECH regimen includes ABT-263 (20), an inhibitor for Bcl-2 and Bcl-xL, and SAR405 (21), an autophagy inhibitor, in combination with LRAs, ingenol-3,20-dibeozoate (IDB) and JQ1 (22, 23). Although SECH treatments can eliminate HIV-infected cells in all blood samples from PLWH *ex vivo* that we have tested so far, it is only effective in clearing HIV infection in 40-77% of humanized mice *in vivo* after 35 cycles (70 days) of treatments (17, 19). Improving the success rate for HIV clearance by SECH *in vivo*, and reducing the cycles of SECH treatments required for viral clearance, will be critical for developing this method as a therapeutic approach. It has been suggested that transcriptome and epigenetic changes play an important role in maintaining the persistence of HIV reservoirs (24–26). We have therefore performed single cell transcriptome analyses of HIV-infected T cells that escaped the treatments, in order to identify cellular pathways that could be targeted to facilitate the deletion of refractory HIV reservoirs.

## Materials and methods

### HIV-1 infection and cure studies using humanized mice

NSG-SGM3 mice were reconstituted with CD34^+^ human stem cells to generate human CD34^+^ stem cell-reconstituted (Hu-HSC) mice for HIV infection and cure studies as described (17). The peripheral blood of mice were analyzed for human immune cells at 10 weeks after reconstitution with human CD34^+^ human stem cells with the following antibodies: Pacific Blue-anti-mouse CD45 (1:100, 103126, Biolegend), APC-anti-human CD45 (1:100, 304012, Biolegend), PerCP/Cyanine5.5-anti-human CD19 (1:100, 302230, Biolegend), Pacific Blue-anti-human CD19 (1:100, 302232, Biolegend), PE/Cyanine7-anti-human CD4 (1:100, 317414, Biolegend), APC/Fire750-anti-human CD8 (1:100, 344746, Biolegend), Pacific Blue-anti-human CD3 (1:100, 300329, Biolegend), PerCP/Cyanine5.5-anti-human CD3 (1:100, 300328, Biolegend) and PE-anti-human CD3 (1:100, 555333, BD Biosciences). Three months after reconstitution with human CD34^+^ stem cells, Hu-HSC mice were infected with HIV-1 AD8 (NIH AIDS Reagent Program, 1000 pfu/mouse, i.p.). Ten days after infection, Hu-HSC mice were given daily suppressive ART treatments with raltegravir (20 mg/kg b.w., Adooq Bioscience), BMS-663068 (20 mg/kg b.w., Adooq Bioscience) and Lamivudine (25 mg/kg b.w., Macleods Pharma.) orally. After 40 days of suppressive ART, the treatments were changed to SECH. For SECH treatments, IDB (2 mg/kg b.w., ENZO Life Sciences), ABT-263 (40 mg/kg b.w., MedChemExpress), SAR405 (40 mg/kg b.w., MedChemExpress) with JQ1 (35 mg/kg b.w., MedChemExpress), together with raltegravir (20 mg/kg b.w.), BMS-663068 (20 mg/kg b.w.), were formulated in the solvent containing 10% ethanol, 30% polyethylene glycol 400 (Sigma), and 60% Phosal 50 PG (Fisher Scientific), and administered by oral gavage once every 2 days. Raltegravir and BMS-663068 (20 mg/kg b.w.) alone were also administered on the alternate days. For the ART control group, raltegravir and BMS-663068 (20 mg/kg b.w.) were given daily. Tablets with non-steroid anti-inflammatory carprofen (2 mg in 5 g tablet, Bio-Serv) were supplied with regular diet to the mice. After 35 cycles of treatments by SECH or ART control, mice were kept for 2 months with no treatments to determine virus rebound as described (17). HIV-1 mRNA in the blood was quantified by RT-PCR according to our established protocols (17). To isolate spleen cells, the spleen from humanized mice were minced and red blood cells were removed by ammonium chloride buffer (0.15 M NH4Cl, 10 mM KHCO3, 0.1 mM EDTA).

### Virus outgrowth assay

TZM-bl cells obtained from the NIH AIDS Reagent Program were cultured in 96 well plates (60,000 cells/well) for 24 hours. Spleen cells (10^6^ cells/well) from Hu-HSC mice were stimulated with anti-CD3- and anti-D28-Dynabeads for 48 h, followed by co-cultured with TZM-bl cells for another 48 h in the presence of 5 μg/ml PHA, 0.1 μg/ml LPS and 100 nM CpG. Beta-galactosidase activity was determined using the Beta-Glo Assay System (Promega). The virus titers in the samples were calculated based on HIV-1 standard titration.

### T cell *in vitro* culture

CD4^+^ T cells sorted from human blood mononuclear cells (PBMC) were infect with HIV-1 (NL4-3, NIH AIDS Reagent Program, 1 MOI), and cultured for 4 days in the presence of CCL19 (30 nM, 582102, Biolegend) and IL-2 (0.3 ng/ml, 589102, Biolegend) to establish latent HIV infection as described (17). To determine the significance of BRD4 in HIV latency reversal and cell killing, latently infected CD4^+^ T cells were treated for 48 h with 3 μM JQ1 or 1 μM MS417 (HY-111139, MedChemExpress), respectively, or with 50 nM ABT-263 and 2 μM SAR405. The cells were then incubated with FITC-DEVD-FMK (1 μM, ab285397, Abcam) at 37 °C for 30 min, followed by staining with APC-Annexin V (1:50, 640941, Biolegend) and analysis by flow cytometry. The percentage of cell death was calculated by the loss of viable cells negative for Annexin V and DEVD staining: (untreated – treated)/untreated x 100%. To determine the significance of glycolysis pathway, CD4^+^ T cells with or without latent HIV-1-infection were treated for 48 h with 80 nM IDB and 50 nM ABT263 together with 15 μM Z57346765 (HY-W195984, MedChemExpress) or 15 μM CBR-470-1 (HY-134205A, MedChemExpress). The cells were then labeled by FITC-DEVD-FMK and stained with APC-Annexin V followed by flow cytometry analysis.

ON-TARGETplus siRNA against human KDM4B (L-004290-00-0005), KDM5C (L-010097-01-0005), PRMT2 (L-004033-00-0005), BRD2 (L-004935-00-0005), BRD4 (L-004937-00-0005), BRDT (L-004938-00-0005) and ON-TARGETplus Non-targeting Pool (D-001810-10-05) were (Horizon Discovery) and transfected individually into CD4^+^ T cells with or without latent HIV-1 infection by Neon Transfection System (ThermoFisher) with 1,700V pulse voltage, 20ms pulse width and 1 pulse number. After 48 h culture, the cells were collected to prepare RNA for quantification of HIV-1 mRNA by RT-PCR. The cells with gene silencing were also treated by 50 nM ABT-263 and 2 μM SAR405 for 48 h and cell death was determined by FITC-DEVD-FMK labelling and APC-Annexin V staining, followed by flow cytometry analysis.

### Treatment of CD4^+^ T cells from HIV-1 patients

Experiments using de-identified samples from HIV patients were performed according to federal and institutional guidelines, with the approval of the Institutional Review Board of the Houston Methodist Research Institute. CD4^+^ T cells were sorted and cultured in RPMI complete medium containing and 5 ng/ml IL-2. The cells were cultured with SECH regimens containing 25 nM IDB, 20 nM ABT-263, 0.1 μM SAR405 and 0.25 μM JQ1, together with 0.2 μM BMS-626529. Only BMS-626529 and raltegravir were added in the ART control group. The cells were cultured for 2 days as one cycle of treatments, washed and cultured in the same medium for next cycle of culture. After 6 cycles (12 days) of treatments, CD3^+^CD4^+^ T cells were sorted by flow cytometry, followed by RT-PCR analyses of HIV-1 mRNA and ChIP-PCR for LTR binding by BRD4.

### Immunohistochemistry staining

Sections of the liver and the spleen cells added on slides by cytospin were incubated with PE-mouse anti-p24 antibody (1:50, 6604667, Beckman Coulter), Alexa Fluor 488-rabbit anti-LC3A/B antibody (1:100, 13082s, Cell Signaling Technology) and Alexa Fluor 647-rabbit anti-human CD4 Antibody (1:50, FAB379R, R&D System) as indicated. The slides were examined using an Olympus FV3000 confocal microscope with the UPLSAPO 100XS objective lens with 100 X magnification and 1.35 numerical aperture, and images were acquired and processed with FV31S-SW software.

### Single cell RNA sequencing and analysis

Human CD4^+^ T cells were sorted by a BD FACSAria flow cytometer (BD Bioscience) from spleen cells of Hu-HSC mice after ART or SECH treatment. Six ART-treated, seven SECH-treated HIV-1^+^ and seven SECH-treated HIV-1^–^ mice were used for scRNA-seq in two independent experiments. Sorted CD4^+^ T cells were used for scRNA-seq analysis at the Single Cell Genomics Core and sequenced at the Genomic and RNA Profiling Core of the Baylor College Medicine.

A custom reference genome was constructed by integrating the human genome (GRCh38) with the sequence of Gag gene of HIV-1 AD8 strain. Sequence alignment was conducted using Cell Ranger (cellranger-8.0.0, 10X Genomics). Cells expressing at least one count of the Gag gene were identified as HIV Gag^+^ cells for subsequent analysis. Datasets were integrated using Canonical Correlation Analysis (CCA) to correct for batch effects. We performed scRNA-seq analyses of 14,771, 14,433 and 8,591 CD4^+^ T cells from ART-HIV^+^, SECH-HIV^+^ and SECH-HIV^−^ mice, respectively. This identification was conducted using feature plots and dot plots with specific gene markers. Clusters were annotated by synthesizing differentially expressed genes (DEGs) identified via Seurat’s FindAllMarkers function (FDR-adjusted p-values using Bonferroni correction). For functional characterization, canonical pathway analysis was conducted to identify enriched biological processes. Chord plots were used to map interactions between pathways and associated DEGs, heat maps to capture expression dynamics, and ridge plots to delineate pathway density between groups. Differential gene expression patterns were further contextualized through volcano plots highlighting DEGs’ significance and fold-change relationships.

### Chromatin immunoprecipitation

Human CD4^+^ cells were enriched by human CD4 MicroBeads (Miltenyi Biotec) from spleen cells isolated form Hu-HSC mice treated with ART or SECH and then cells were cross-linked with 1% formaldehyde for 10 min at room temperature. The cells were lysed on ice for 10 min by SDS Lysis buffer (1% SDS, 10 mM EDTA, 50 mM Tris-HCl pH 8). The lysate was then sonicated for 60 cycles at 50% amplitude with a sonicator (Q800R3, Qsonica) to shear chromatin to 200-400bp. A fraction (1/30 of volume) of sheared chromatin was used as input sample, and the rest was incubated with anti-BRD4 (Cell Signaling, Cat. No. 13440S) and Dynabeads Protein G beads (ThermoFisher) at 4°C overnight. After incubation, DNA was eluted from beads with the elution buffer (100 mM NaHCO3, 1% SDS). Both input and eluted DNA were reversed cross-linking at 65°C with shaking in the presence of 400 mM NaCl and proteinase K (0.2 μg/ml). DNA was purified by phenol/chloroform extraction and followed by PCR to amply the 5’LTR of HIV-1. The primers for 5’LTR of HIV-1 are as follows: BRD4-ChIP-LTR-F, 5’-TCTACAAGGGACTTTCCGCT-3’ and BRD4-ChIP-LTR-R, 5’-TGAGGGATCTCTAGTTACCAGAGTC-3’. CD4^+^ T cells sorted from PBMCs of five ART-experienced patients were treated with ART or SECH for 6 cycles, followed by ChIP assay as described above. CD4^+^ cells purified from PBMCs were latently infected with HIV-1 (NL4-3) as described above and treated with or without JQ1 (10 μM) for 18h followed by ChIP assay.

### Statistical analyses

GraphPad Prism (version 10.2.1) was used for statistical analyses of biological assays. Data were presented as the mean ± SD. *P* values were determined by two-tailed Student’s t-test or one-way ANVOA with unpaired two-tailed t test. Significant statistical differences (*P*< 0.05 or *P*< 0.01) are indicated.

## Results

### Differential gene expression in HIV reservoirs resistant to depletion

**Figure 1:**
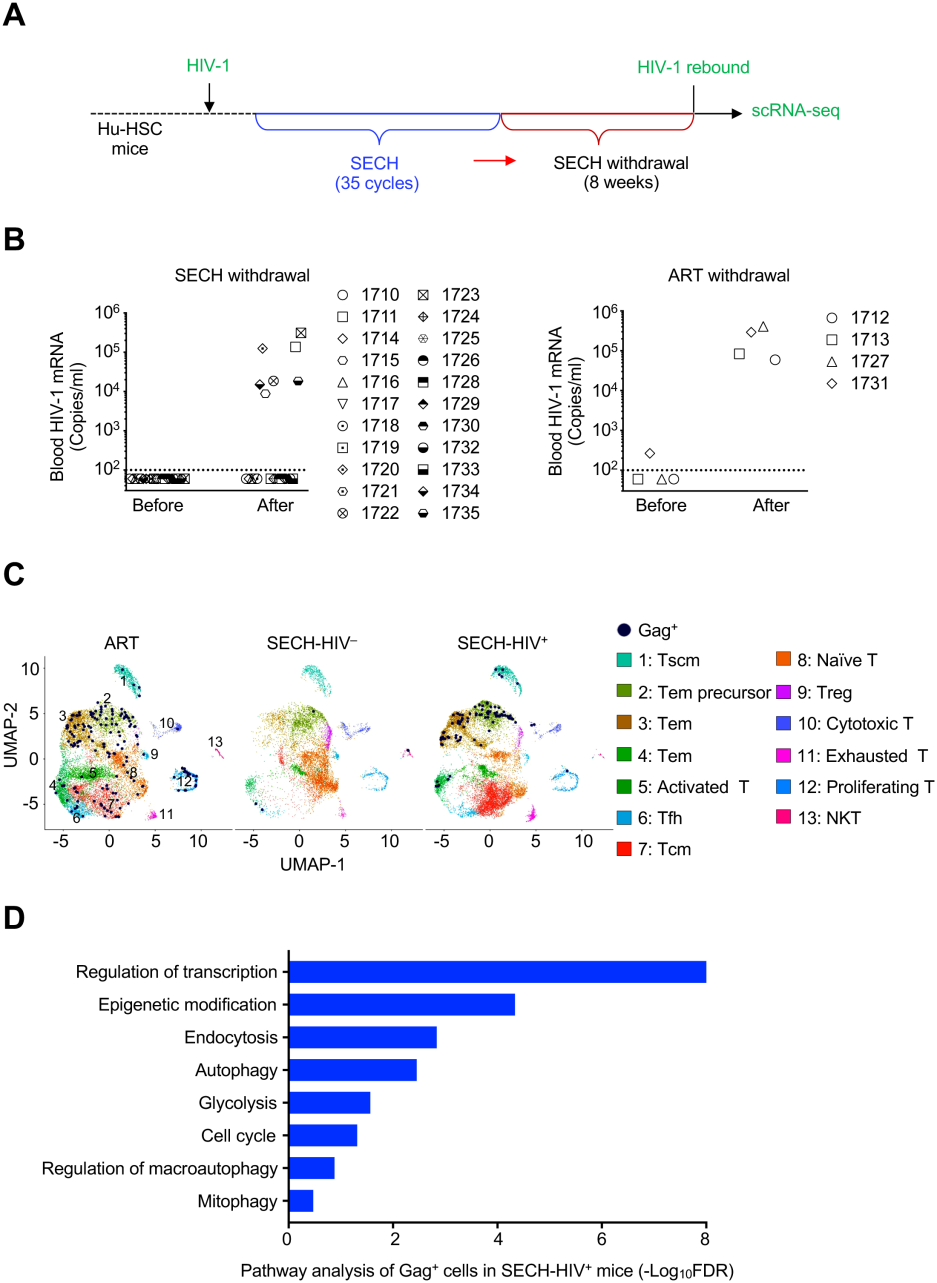
Single cell transcriptome analysis of CD4+ T cells from HIV-1 infected Hu-HSC mice treated by SECH or ART. **(A)** A schematic diagram of SECH regimen. Hu-HSC mice were infected with HIV-1 AD8 and treated with suppressive ART for 40 days to establish latent infection. The mice were then treated for 35 cycles by SECH regimens containing IDB, JQ1, ABT-263 and SAR405 together with ART, or by ART only, followed by 2 months of drug withdraw to determine HIV rebound. **(B)** HIV-1 viremia was determined before and after 2 months of withdrawal of SECH (Left panel) or ART (Right panel). **(C)** scRNA-seq analyses of CD4^+^ T cells from Hu-HSC mice treated by SECH with success (SECH-HIV^–^) or failure (SECH-HIV^+^) in HIV clearance, or treated by ART control. Thirteen clusters (1–13) in CD4^+^ T cells from Hu-HSC mice treated with ART or SECH were identified by Uniform Manifold Approximation and Projection (UMAP) analysis and visualized with distinct color scheme. Gag^+^ T cells were highlighted by dark blue dots. **(D)** Enrichment of cell signaling pathways in Gag^+^ CD4^+^ T cells from SECH-HIV^+^ mice compared to CD4^+^ T cells in SECH-HIV^–^ mice.

By targeting pro-survival autophagy and anti-apoptotic molecules during HIV reactivation, the SECH approach could clear HIV infection in peripheral blood mononuclear cells (PBMCs) from PLWH (17). In the *in vivo* setting, the SECH treatments led to the clearance of HIV infection only in a portion of human CD34^+^ stem cell-reconstituted (Hu-HSC) mice after 35–40 cycles of treatments (17). We set out to determine the mechanisms for the resistance of HIV reservoirs to SECH treatments in humanized mice. To achieve this goal, we established a new batch of Hu-HSC mouse model by engraftment of human CD34^+^ stem cells into NSG-SGM3 mice. After stem cell implantation, the mice were efficiently reconstituted with human CD4^+^ and CD8^+^ T cells, B cells, dendritic cells and macrophages (**Figure 1A**, **Supplementary Figures S1A, B**). TCR sequencing revealed the variety of Vβ gene segment usage, suggesting the diversity of the T cell population (**Supplementary Figure S1C**). These mice were then infected with HIV-1 and treated with suppressive ART, following with SECH approach (**Figure 1**). Similar to previous findings (17), we did not observe signs of inflammation or tissue injuries by SECH treatments. However, SECH treatments led to a decrease in naïve T cels and an increased in effector/memory T cells (**Supplementary Figure S1D**), suggesting certain degree of T cell activation. We found that 69% of latently infected Hu-HSC mice were free of HIV-1 infection after SECH treatments, as shown by the lack of viral rebound after withdrawal of treatments (**Figure 1B**). However, 31% Hu-HSC mice displayed HIV rebound after withdrawal of SECH treatments (**Figure 1B**), suggesting that these mice are resistant to SECH treatments. In comparison, all mice treated by ART alone showed viral rebound after ART withdrawal (**Figure 1B**). Consistently, infectious HIV-1 was detected by virus outgrowth assay in the spleen of the mice with viral rebound after SECH treatments, or in control mice treated by ART (**Supplementary Figure S2A**). In contrast, infectious HIV-1 was not found in mice with no HIV rebound after withdrawal of SECH treatments (**Supplementary Figure S2A**). We have previously found that lack of HIV rebound is correlated with a reduction of viral DNA (17). This indicates that some HIV reservoirs are refractory to SECH treatments in Hu-HSC mice. It will be very interesting to determine rebound kinetics in these mice and to correlated with reservoir size in the future.

To find out the mechanisms for the resistance of reservoirs from Hu-HSC mice with failed HIV clearance, we performed single cell RNA sequencing (scRNA-seq) analyses of CD4^+^ T cells from these mice (**Supplementary Figure S2B**). Human CD4^+^ T cells were sorted from the spleen of HIV-infected Hu-HSC mice (pooled from 7 mice/group) treated by SECH with success (SECH-HIV^–^) or failure (SECH-HIV^+^) in HIV clearance, or treated by ART control (ART-HIV^+^). Among 14,771 CD4^+^ T cells from ART-treated mice analyzed by scRNA-seq, a total of 165 Gag^+^ T cells were found in UMAP cell clusters (**Figure 1C**). In the analyses of 14,433 T cells from SECH-HIV^+^ mice, we detected 88 Gag^+^ T cells (**Figure 1C**). Pathway analyses show that genes involved in autophagy, glycolysis and epigenetic modification were elevated in Gag^+^ T cells from SECH-HIV^+^ mice (**Figure 1D**). Interestingly, these Gag^+^ T cells from SECH-HIV^+^ mice were enriched in clusters 1–4 that contain T effector memory (Tem), Tem precursors and stem central memory T (Tscm) cells (**Figure 1C**, **Supplementary Figure S2C**). This suggests that T cell reservoirs resistant to SECH treatments are enriched in Tem and Tscm cells.

### Elevated autophagy in SECH-resistant T cell reservoirs

**Figure 2:**
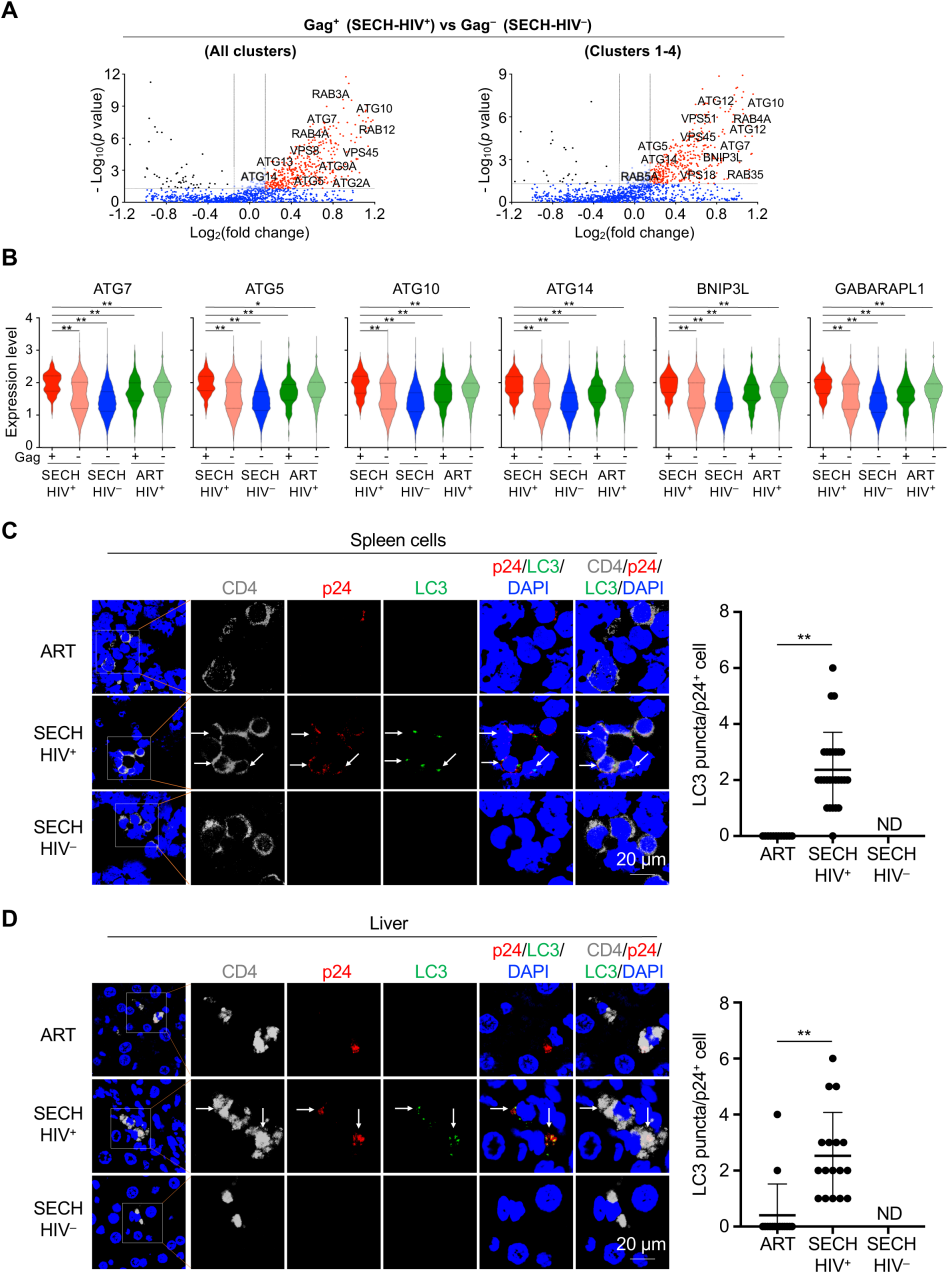
Enhancement of autophagy in Gag+ CD4+ T cells by SECH treatment. **(A)** Volcano plots for differential expression of autophagy genes in Gag^+^ CD4^+^ T cells from SECH-HIV^+^ mice comparing to CD4^+^ T from SECH-HIV^–^ mice. **(B)** Violin plots for the expression of autophagy genes in Gag^+^ or Gag^–^ CD4^+^ T cells in humanized mice with failed HIV clearance by SECH (SECH-HIV^+^), Gag^–^ CD4^+^ T cells with successful HIV clearance (SECH-HIV^–^), and Gag^+^ or Gag^–^ CD4^+^ cells from mice treated by ART control. **P<0.01. **(C, D)** Immunocytochemistry staining for human CD4 (grey), HIV-1 p24 (red) and LC3 (green) in the spleen **(C)** and liver **(D)** from humanized mice treated by by SECH with success (SECH-HIV^–^) or failure (SECH-HIV^+^) in HIV clearance, or treated ART control. The nuclei were stained with DAPI. The sections were examined by confocal microscopy. Arrows indicate p24 and LC3 staining. **P<0.01. ND, p24 staining not detected.

Autophagy plays an important role in the maintenance of long-lived immune memory cells (27–30). Increased autophagy in Gag^+^ T cells from SECH-HIV^+^ mice may contribute to the resistance to SECH-induced deletion. We found increased expression of genes involved in autophagy in Gag^+^ T cells from SECH-HIV^+^ mice, including ATG5, ATG7, ATG9A, ATG10, ATG12 and ATG14 in general autophagy, NIX in mitochondrial autophagy (31), as well as RAB3A, RAB4A and RAB12 involved in vehicle sorting for autophagosome formation (32, 33) by volcano plot (**Figure 2A**, left panel), violin plot (**Figure 2B**) or bubble plot (**Supplementary Figure S3**) analyses. The expression of autophagy genes in Gag^+^ T cells of memory precursor or effector memory T cells of clusters 1–4 from SECH-HIV^+^ mice were also higher than in the same cells of these clusters from SECH-HIV^–^ mice (**Figure 2A**, right panel). Increased expression of autophagy genes in Gag^+^ T cells from SECH-HIV^+^ mice was also observed by comparison with Gag^–^ T cells from the same SECH-HIV^+^ mice, or with Gag^+^ and Gag^–^ T cells from mice treated by ART control (**Figure 2B**, **Supplementary Figure S3**). These results suggest that Gag^+^ T cells from SECH-treated mice with failed HIV clearance express elevated levels of autophagy genes.

To confirm the finding by scRNA-seq, we performed immunocytochemistry staining to detect autophagy in T cells of Hu-HSC mice resistant to SECH treatments. In the spleen of SECH-treated Hu-HSC mice with unsuccessful HIV-1 clearance (SECH-HIV^+^), p24^+^ T cells displayed increased LC3 punctate staining (34) characteristic of active autophagy (**Figure 2C**). In comparison, p24^+^ T cells from mice treated by ART control showed significant less LC3 staining (**Figure 2C**). Similar increases in LC3 staining were observed in p24^+^ T cells in the liver of Hu-HSC mice resistant to SECH treatments (**Figure 2D**). Because autophagy is a main target for inhibition by SECH (17), these results support the possibility that reservoirs with elevated autophagy are more likely to escape the induction of cell death by SECH treatments.

### Increased glycolysis pathways in T cell reservoirs resistant to SECH treatments

**Figure 3:**
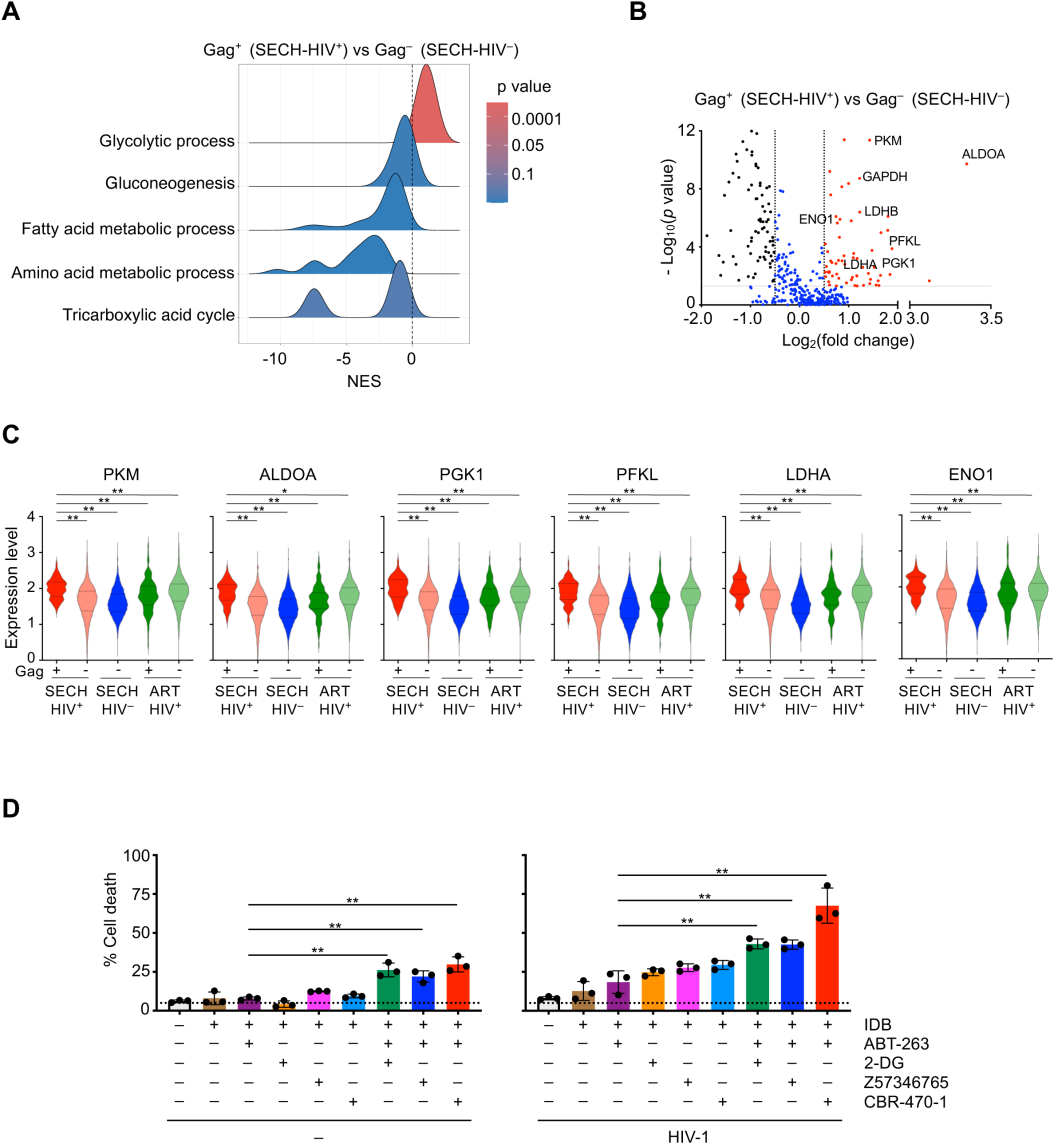
Augmentation of glycolysis in Gag+ CD4+ T cells resistant to SECH. **(A)** Ridgeline plot shows that glycolysis pathway was enriched in Gag^+^ CD4^+^ T cells from Hu-HSC mice with failed SECH treatments (SECH-HIV^+^) comparing with Gag^–^ CD4^+^ cells in mice with successful SECH treatments (SECH-HIV^–^). **(B)** Volcano plots for the expression of glycolytic genes in Gag^+^ T cells from the Hu-HSC mice with failed SECH treatments (SECH-HIV^+^) compared with Gag^–^ T cells from the mice with successful SECH treatments (SECH-HIV^–^). **(C)** Violin plots for the expression of glycolytic genes in Gag^+^ or Gag^–^ CD4^+^ T cells from Hu-HSC mice with failed SECH treatments (SECH-HIV^+^), Gag^–^ CD4^+^ T cells in mice with successful SECH treatments (SECH-HIV^–^) and Gag^+^ or Gag^–^ CD4^+^ T cells in mice treated by ART control. **P<0.01. **(D)** Latently infected CD4^+^ T cells were treated with IDB, ABT-263, and 2-DG or PGK1 inhibitors, Z57346765 and CBR-470-1, as indicated. Cell death was determined by DEV-FITC labelling and annexin V staining followed by flow cytometry analysis. **P<0.01.

Among different metabolic pathways, glycolysis is most significantly upregulated in Gag^+^ T cells resistant to SECH treatments (**Figure 3A**). Genes involved in glycolysis, including PKM, ALDOC, PGK1, PFKL, LDHA and ENO1 (35), were significantly increased in Gag^+^ cells from SECH-HIV^+^ mice compared to Gag^–^ cells from SECH-HIV^–^ mice (**Figures 3A–C**). These genes were also increased in these Gag^+^ T cells compared to Gag^–^ cells of the same SECH-resistant (SECH-HIV^+^) mice, or with Gag^+^ and Gag^–^ T cells from mice treated by ART control, as shown by violin plot (**Figure 3C**) and bubble plot **(Supplementary Figure S4)** analyses. These results indicate that the glycolysis pathway is more active in HIV-infected T cells from mice resistant to SECH treatments.

We next examined whether inhibition of glycolysis could sensitize HIV-infected T cells to undergo cell death. Treatments with 2-deoxy-D-glucose (2-DG), a glucose analog as a glycolysis inhibitor (36), increased cell death in HIV-1-infected T cells in the presence of SECH components, IDB and ABT-263 (**Figure 3D**, right panel). However, 2-DG also increased cell death in uninfected cells under this condition (**Figure 3D**, left panel). Similar results were observed using pyruvate dehydrogenase kinase 1 (PGK1) inhibitors, Z57346765 or CBR-470-1 (37, 38), to target glycolysis (**Figure 3D**). These observations suggest that inhibition of glycolysis can sensitize cell death not only in HIV-infected T cells, but also in uninfected cells. Therefore, the glycolysis pathway is not a suitable target for suppression to promote selective killing of HIV-infected T cells.

### Increased epigenetic regulators in reservoirs resistant to SECH treatments

**Figure 4:**
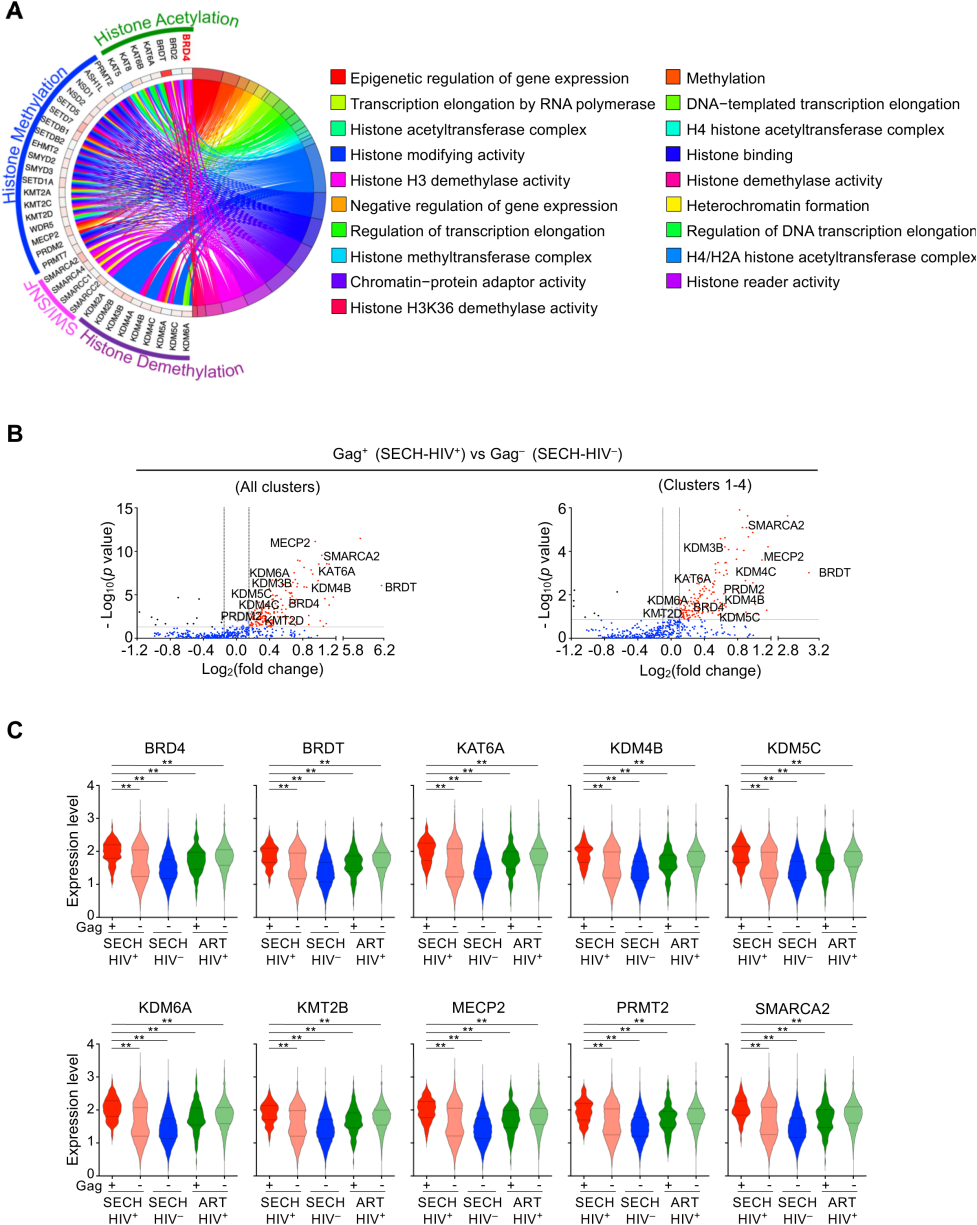
Elevated epigenetic signaling in Gag+ CD4+ T cells resistant to SECH. **(A)** Circular plot for elevated expression of the genes involved in epigenetic regulation in Gag^+^ T cells from the Hu-HSC mice with failed SECH treatments (SECH-HIV^+^) compared with Gag^–^ T cells in mice with successful SECH treatments (SECH-HIV^–^). **(B)** Volcano plot for the expression of epigenetic regulators in Gag^+^ T cells from the Hu-HSC mice with failed SECH treatments (SECH-HIV^+^) compared with Gag^–^ T cells in mice with successful SECH treatments (SECH-HIV^–^). **(C)** Violin plots for gene expression of epigenetic modifiers in Gag^+^ or Gag^–^ T cells from Hu-HSC mice with failed SECH treatments (HIV^+^), Gag^–^ T cells in mice with successful SECH treatments (SECH-HIV^–^) and Gag^+^ or Gag^–^ T cells in mice treated by ART control. **P<0.01.

We observed upregulation of epigenetic modifiers in Gag^+^ T cells resistant to SECH treatments (SECH-HIV^+^) compared with Gag^–^ T cells from SECH-HIV^–^ mice (**Figures 4A, B).** These genes include BRD4 and BRDT of the bromodomain and extra-terminal domain (BET) family proteins (39), KDM4B, KDM5C and KDM6A of the histone lysine demethylases (KDM) family members (40), regulators of protein methylation, including MECP2, KMT2B and PRMT2 (41–43), and histone acetyltransferase KAT6A (44). The increases in these epigenetic modifiers in Gag^+^ T cells from SECH-treated mice with failed HIV clearance (SECH-HIV^+^) were also observed when compared with Gag^–^ T cells from the same mice, or with Gag^+^ and Gag^–^ T cells from mice treated by ART control (**Figure 4C**). Among the genes upregulated in SECH-resistant Gag^+^ T cells, BRD4 is a histone modifier that negatively regulate of HIV gene expression (45, 46). PRMT2, a histone methyltransferase increased in SECH-resistant Gag^+^ T cells, can inhibit HIV-1 gene transcription at the elongation stages (42). It is possible that increased levels of epigenetic modifiers suppress HIV gene expression, thereby enabling HIV-infected T cells to escape depletion during SECH treatments.

### Targeting epigenetic modifiers in HIV-infected cells

**Figure 5:**
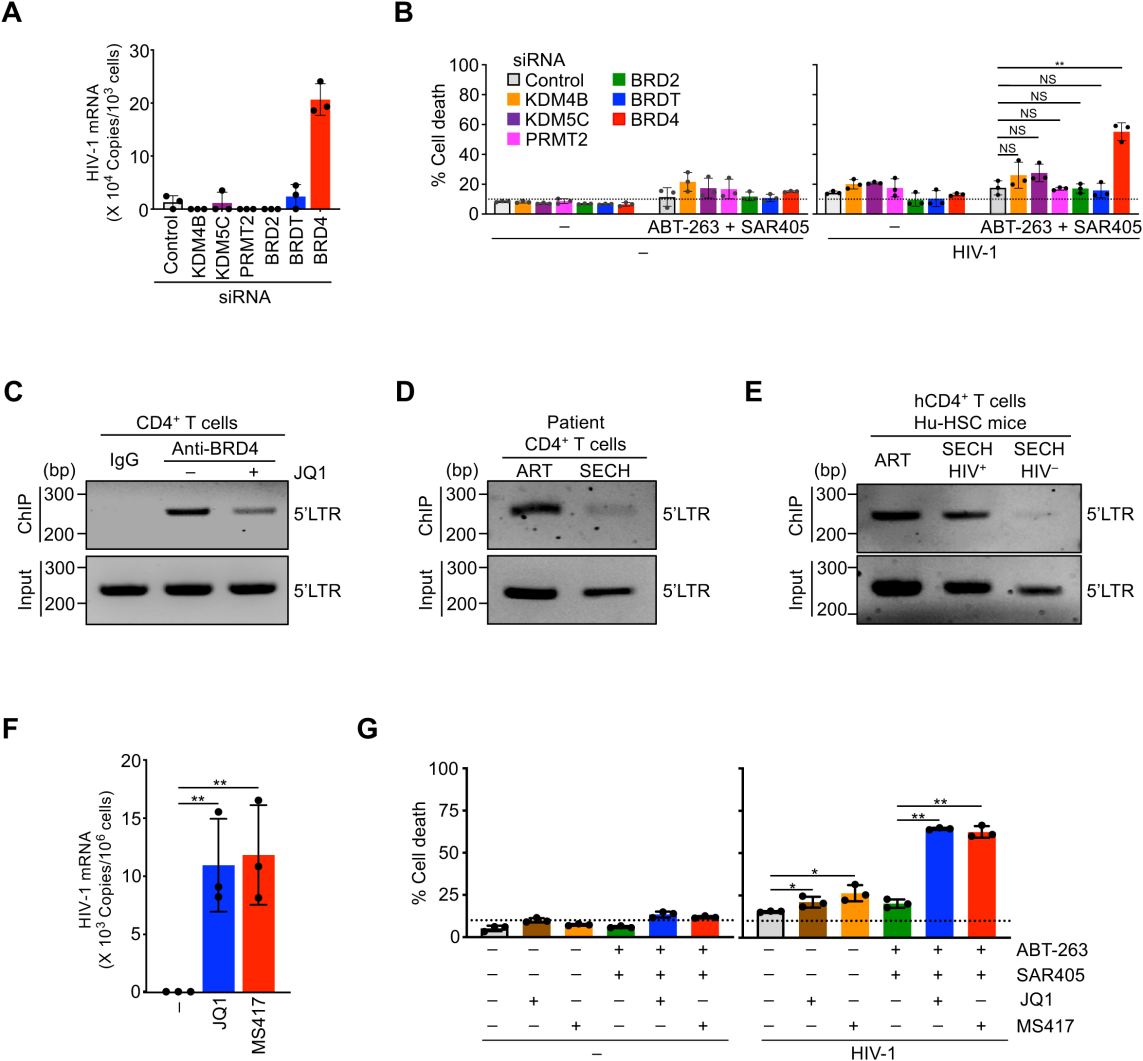
Sensitization of HIV-1-infected CD4+ T cells to cell death by targeting epigenetic modifiers. **(A)** HIV-1 reactivation in CD4^+^ T cells with latent HIV-1 infection by silencing epigenetic modifiers. **P<0.01. **(B)** Promotion of cell death in CD4^+^ T cells with latent HIV-1 infection by silencing of epigenetic modifiers. **P<0.01. **(C)** ChIP-PCR analysis for the binding of BRD4 to HIV-1 LTR in CD4^+^ T cells latently infected by HIV-1 with or without JQ1 treatment. **(D)** ChIP-PCR analysis for the binding of BRD4 to HIV-1 LTR in CD4^+^ T cells from patient PBMCs after ART or SECH treatments. The treated samples from 4 patients were pooled for ChIP-PCR analysis. **(E)** ChIP-PCR analysis for BRD4 binding to HIV-1 LTR in CD4^+^ T cells from humanized mice treated by ART, or by SECH with success (SECH-HIV^–^) or failure (SECH-HIV^+^) in HIV clearance. The cells from 3 mice in each group were pooled for ChIP-PCR. **(F)** Induction of HIV-1 mRNA expression in CD4^+^ T cells latently infected by HIV-1 aftre treatments with BRD4 Inhibitors. **P<0.01. **(G)** Promotion of cell death in CD4^+^ T cells with latent HIV-1 infection by BRD4 inhibitors **P<0.01.

To determine whether changes in these epigenetic modifiers results in reduced HIV gene expression and subsequence resistance to clearance, we examined the effects of silencing these genes in HIV-1-infected T cells (**Supplementary Figure S5**). While increases in KDM4B, KDM5C, PRMT2, BRD2 and BRDT were found in SECH-resistant Gag^+^ T cells, the silencing of these genes did not promote viral gene expression (**Figure 5A**), or increase the killing of HIV-1-infected T cells (**Figure 5B**). In contrast, silencing of BRD4 induced viral gene expression in HIV-infected T cells (**Figure 5A**). Moreover, silencing of BRD4 facilitated the killing of HIV-infected T cells in the presence of ABT-263 and SAR405 (**Figure 5B**). These results suggest that BRD4 can be targeted to promote HIV gene expression and facilitate the killing of HIV-infected cells.

BRD4 may interact with acetylated histones of chromatin in the promoter regions of target genes to promote gene expression (47). To determine the potential interactions of BRD4 with HIV-1 LTR, we performed chromatin immunoprecipitation (ChIP) analysis to determine the association of BRD4 with HIV-1 LTR. In T cells with latent HIV-1 infection, we could detect the binding of BRD4 to HIV-1 LTR by ChIP-PCR (**Figure 5C**). A competitive inhibitor for BRD4, JQ1 (48), decreased the binding of BRD4 to HIV-1 LTR (**Figure 5C**), suggesting that HIV-1 LTR is occupied by BRD4 in T cells with latent HIV-1 infection.

We next examined the interaction of HIV-1 LTR with BRD4 in patient CD4^+^ T cells (**Supplementary Figure S6**). In patient T cells treated by ART control, we detected the binding of BRD4 with HIV-1 LTR by ChIP-PCR (**Figure 5D**). This indicates that viral LTR is occupied by BRD4 in patient T cells. We further examined how SECH treatments could affect the interaction of HIV-1 LTR with BRD4. While SECH treatments could clear infectious HIV-1 in patient T cells (17), HIV-1 LTR was reduced by still detectable in the total chromatin input from patient T cells treated by SECH (**Figure 5D**). This is consistent with our previous observation of a decrease in HIV-1 proviral DNA after HIV clearance by SECH treatments (17).

The remaining viral DNA in SECH-treated samples likely represents non-productive HIV proviruses that are accumulated during viral infection. It has been shown that only a small portion of integrated HIV-1 proviruses is intact and infectious, while most HIV-1 proviruses in infected cells are defective (49). Because the SECH approach depends on the induction of cytopathic viral genes from intact HIV to induce cell death (17), the cells with defective HIV-1 proviruses that produce little or no HIV gene products would not be deleted by SECH treatments. In an HIV-1 patient free of the infectious virus after treatment by stem cell transplantation, residual HIV DNA can be detected in the tissues (50), consistent with the possibility that defective HIV-1 can still exist after the clearance of infectious HIV-1. In patient T cells treated by ART control, we also detected the interactions between BRD4 and HIV-1 LTR (**Figure 5D**). These results indicate that HIV-1 LTR is occupied by BRD4 in patient T cell reservoirs. In these patient T cells treated by SECH with successful clearance of infectious HIV-1, the interaction between BRD4 and HIV-1 LTR was significantly reduced as shown by ChIP-PCR (**Figure 5D**), indicating that SECH treatment can significantly reduce HIV-1 LTR occupied by BRD4.

We next performed ChIP-PCR analyses to examine the interaction of HIV-1 LTR with BRD4 in SECH treated Hu-HSC mice. Similar to above observations with patient T cells, HIV-1 LTR was reduced but still present in total chromatin input from T cells of Hu-HSC mice after SECH treatments (**Figure 5E**), consistent with the possibility that SECH is effective in clearing intact but not defective HIV-1 proviruses. In T cells from Hu-HSC mice treated by ART, or treated by SECH with failed HIV clearance (SECH-HIV^+^), we could detect the binding of BRD4 to HIV-1 LTR (**Figure 5E**). In contrast, the association of BRD4 with HIV-1 LTR was significantly reduced in Hu-HSC mice with successful HIV clearance (SECH-HIV^–^; **Figure 5E**). Because SECH-HIV^–^ mice did not have detectable infectious HIV-1, the residual HIV-1 LTR associated with BRD4 is potentially from defective proviruses. Together, these results suggest that the HIV-1 LTR in SECH-resistant reservoirs is occupied by BRD4.

It has been shown that inhibition of BRD4 promotes the reactivation of HIV-1 (45, 46). Consistent with the effects of targeting BRD4 by siRNAs, BRD4 inhibitor JQ1 or MS417 (51) induced HIV-1 reactivation in infected T cells and triggered cell death in HIV-1-infected T cells (**Figure 5, F** and G). Moreover, JQ1 or MS417 increased the killing of HIV-1-infected T cells in the presence of SECH components, ABT-263 and SAR405 (**Figure 5G**). Together, these data suggest that targeting BRD4 is critical for efficiently inducing HIV-1 reactivation to promote cell death of HIV-1-infected cells.

### Resistance to SECH treatments by HIV reservoirs in cell clusters enriched with effector memory and stem memory T cells

**Figure 6:**
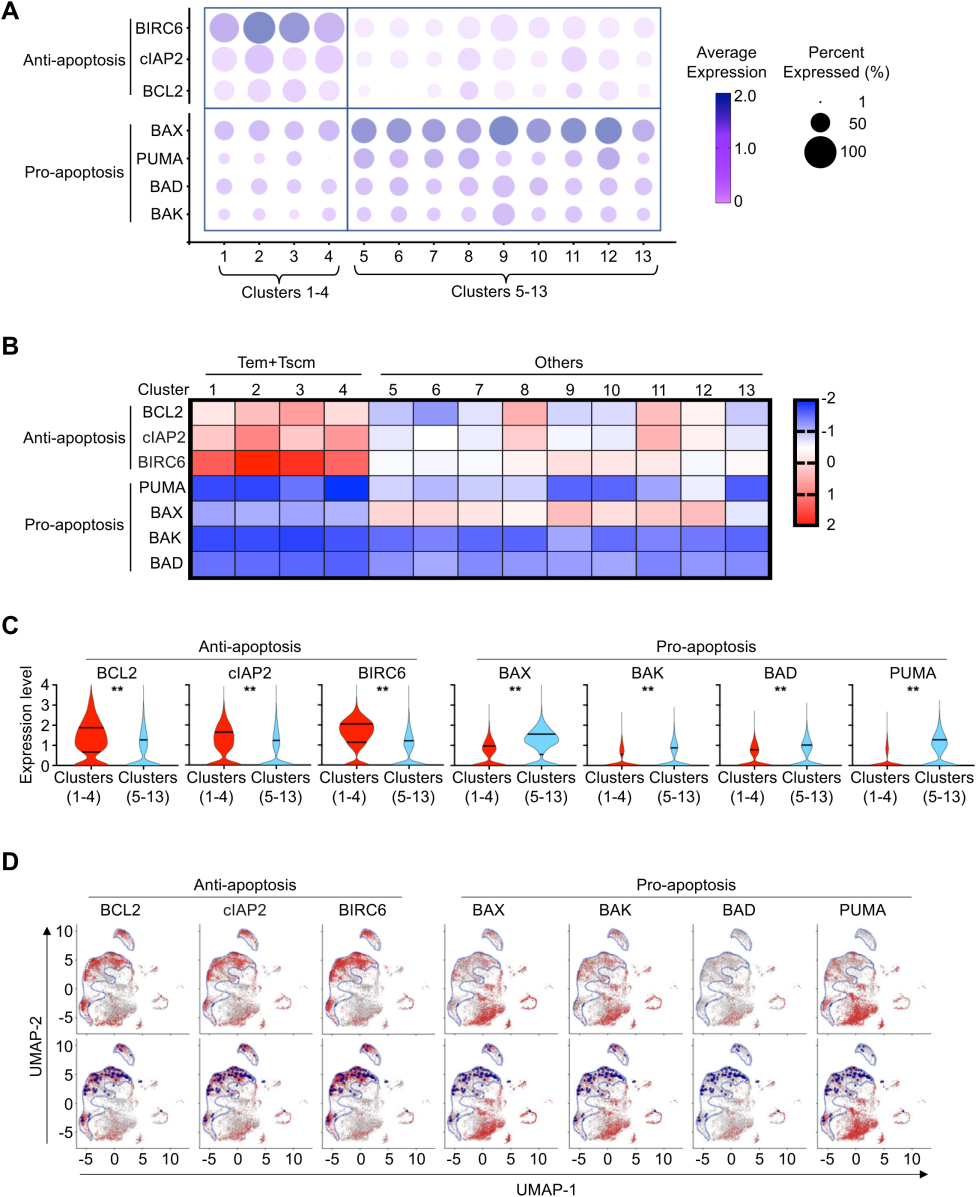
Resistant to cell death of CD4+ T cells in clusters 1-4. **(A)** Bubble plot depicts the average level of normalized expression of apoptotic and anti-apoptotic genes in CD4^+^ T cells from each cluster in SECH-treated mice with failure in HIV clearance. The color of bubble represents the average level of normalized expression of the genes of indicated. The size of bubble represents the percentage of cells expressing the genes of indicated. **(B, C)** Differential expression of apoptotic and anti-apoptotic genes in CD4^+^ T cells from cluster 1–4 comparing to T cells from the other clusters (5–13) in SECH-HIV^+^ mice, as shown by heatmap **(B)** and violin plot **(C)**. **P<0.01. **(D)** Feature plots project the normalized expression of apoptotic and anti-apoptotic genes in CD4^+^ T cells from SECH-HIV^+^ mice onto the UMAP. Cluster 1–4 was delineated by blue line in upper panel. Gag^+^ CD4^+^ T cells were also highlighted by dark blue dot on UMAP in lower panel.

Interestingly, a majority of HIV-1-infected T cells resistant to SECH treatments were found in clusters 1–4 enriched in effector memory or stem memory T cells (**Figure 1C**). Preferential retention of Gag^+^ T cells from SECH-HIV^+^ mice in clusters 1–4 of scRNA-seq suggests that molecular pathways in these groups of cells may confer resistance to cell death. Interestingly, T cells in clusters 1–4 show increases in the expression of anti-apoptotic BIRC6, cIAP-2 and BCL-2, as well as decreases in pro-apoptotic BAX, BAK, BAD and PUMA, as shown by bubble plot (**Figure 6A**), heatmap (**Figure 6B**) and violin plot (**Figure 6C**) analyses. UMAP plots show that the expression of HIV-1 Gag is correlated with anti-apoptotic BIRC6, cIAP-2 and BCL-2, but inversely correlated with pro-apoptotic BAX, BAK, BAD and PUMA (**Figure 6D**). These results indicate that HIV reservoirs in T cell subsets with decreased apoptosis are more resistant to SECH-mediated deletion.

## Discussion

In this study, we investigated the mechanisms for HIV-infected T cells to escape depletions induced by SECH treatments. The SECH strategy is designed to sensitize HIV reservoirs to cell death, by counteracting pro-survival autophagy and anti-apoptotic molecules that are up-regulated during HIV reactivation. By single cell transcriptome analyses of HIV-1-infected T cells in humanized mice resistant to SECH treatments, we observed increases in the expression of epigenetic modifiers and autophagy genes. Immunohistochemistry staining confirmed elevated autophagy in HIV-1-p24^+^ T cells resistant to SECH treatments. These results are reminiscent of the selectively reduction of HIV-infected cells by treatments with autophagy inhibitors or Atg7 siRNAs (17). Chromatin immunoprecipitation analyses indicate that HIV-1 LTR in SECH-resistant HIV reservoirs is occupied by BRD4, which can inhibit HIV gene expression and prevent the killing of host cells. Interestingly, SECH-resistant HIV reservoirs were mainly found in cell clusters containing stem memory and effector memory T cell subsets. These T cell clusters showed decreases in pro-apoptotic and increases in anti-apoptotic genes, which likely contributed to the escape of HIV reservoirs in these T cell subsets. T cell reservoirs with increased epigenetic modifiers and autophagy are more likely to survive under the pressure of SECH-mediated killing, leading to their enrichment in mice with failed HIV clearance. Targeting the balance of repressed HIV gene expression and the evasion from cell death may be especially important for eliminating these refractory HIV reservoirs.

High levels of HIV gene expression in host cells is expected to trigger cell death signaling, and promote the recognition and killing by cytotoxic T cells. In contrast, the reservoirs that keep the viral production sufficiently low can escape the cytopathic effect of HIV gene products, and limit viral antigen presentation for immune attack. Therefore, these reservoirs refractory to treatments maintain a delicate balance between controlled viral replication and evasion of cell death, can persist in the patients and continue to spread infections. Interestingly, very low levels of viral gene transcription are found in T cells with latent intact HIV-1 integration (52). Such features of latent reservoirs likely contribute to low levels of viral spread and long-term persistence of HIV reservoirs. In Hu-HSC mice with failed HIV clearance after SECH treatment, HIV-1 p24 is detected in the spleen after withdrawal of SECH (**Figure 2**). Such HIV reservoirs may be able to tolerate low levels of expression of cytopathic HIV genes when the epigenetic regulators keep the HIV gene expression in check. Additionally, higher expression of autophagy genes can also support cell survival in these reservoir cells. Targeting the balances of repressed HIV gene expression and the evasion from cell death will be important for eliminating these refractory reservoirs. The CD34^+^ stem cell-reconstituted Hu-HSC mice used in this study are different from human PBMC-reconstituted (Hu-PBMC) mice. A major limitation of Hu-PBMC model is the inevitable and rapid development of lethal graft-versus-host disease (GVHD) (53), which limits the lifespan of mice. Hu-HSC mice in this study provide efficient reconstitution of human immune system with stable and robust human CD4^+^ and CD8^+^ T cells, B cells, dendritic cells and macrophages (**Supplementary Figure 1**), with no severe GVHD responses. Hu-HSC mice appear to be more suitable for HIV infection and cure studies.

In addition to induce different forms of cell death in latent HIV reservoirs, some LRAs have been shown promote cell survival (54–56). While HIV regulatory proteins can alter the induction of autophagy (57–62), treatments with an LRA, ingenol-3,20-dibenzoate, induced autophagy in T cells with latent HIV infection, and in control T cells without HIV infection (17). These results suggest that LRA stimulation can induce autophagy in the presence or absence of HIV. Interestingly, reactivation of HIV-1 also induces the expression of anti-apoptotic molecules and autophagy in T cells. Mechanistically, the promotors for anti-apoptotic and autophagy genes share the binding sites for transcription factors, such as NF-κB, NFAT, AP-1 and HIF-1α that bind to HIV-1 LTR to drive viral gene expression (18). It is therefore expected that pro-survival autophagy and anti-apoptotic pathways will be induced simultaneously with the activation of HIV-1 promoters (18). It is thus necessary to inhibit the pro-survival mechanisms that can counteract the cytopathic effects of induced HIV genes to sensitize HIV reservoirs to cell death.

Both the cellular transcriptional machinery and the accessibility of HIV-1 proviruses in the host genome are critical for viral reactivation. First, it is important to stimulate host cells to express cellular transcription factors that bind to and activate the HIV-1 promoter in the 5’-LTR (18). Second, the HIV-1 provirus needs to be accessible by the host transcription machinery. Transcriptome analyses revealed increases in the expression of epigenetic modifiers, such as BRD4, in Gag^+^ T cells from Hu-HSC mice resistant to SECH treatments. We have previously shown that a BRD4 inhibitor, JQ1, increased HIV reactivation and HIV clearance (17). Indeed, targeting BRD4 with JQ1 can increase HIV reactivation and promote cell death in HIV-infected T cells, indicating that epigenetic regulators that inhibit HIV reactivation contribute to resistance to SECH treatments in T cells. Therefore, efficient viral reactivation with LRAs, including the targeting of epigenetic factors, is critical for triggering cell death in HIV reservoirs in T cells. We have found that using two different classes of LRAs, including JQ1, an epigenetic modifier, and IDB, a protein kinase C activator, induced better HIV-1 reactivation and viral clearance (17–19). In clinical settings, it will be desirable to use as few agents as possible in a combination therapy. It will be important to develop more powerful LRAs with dual functions that not only activate the cellular transcriptional machinery, but also increase the epigenetic accessibility of HIV proviruses, to improve HIV reactivation for better viral clearance.

The maintenance of viral latency and protection of host from cell death are potentially the major mechanisms for the long-term persistence of HIV reservoirs. This study indicates that persistent HIV-infected T cells may keep a delicate balance to maintain a low level of HIV replication, while managing to evade cell death. The elevated epigenetic repressors for HIV gene expression and increased autophagy may contribute to the escape of HIV-infected T cells from depletion. Other factors, such as the HIV integration sites in the host genome, may contribute to the resistance to viral clearance and should be investigated in the future (63). Targeting various mechanisms for the resistance to deletion will be critical for improving the success rate for the depletion of reservoir cells to clear HIV infection.

## Data Availability

All data supporting the findings of this study are available within the article and are available from the corresponding author. The scRNA-seq data presented in the study are deposited in the NCBI BioProject, accession number PRJNA1285998.
