# Supplementary material for: Promotion of HIV clearance by sensitization of HIV reservoirs to cell death
Source: Front Immunol. 2025 Jul 14;16:1600741. doi: 10.3389/fimmu.2025.1600741 (PMC12302414; doi:10.3389/fimmu.2025.1600741)
Supplement: Supplementary file 1 [file DataSheet1.pdf]

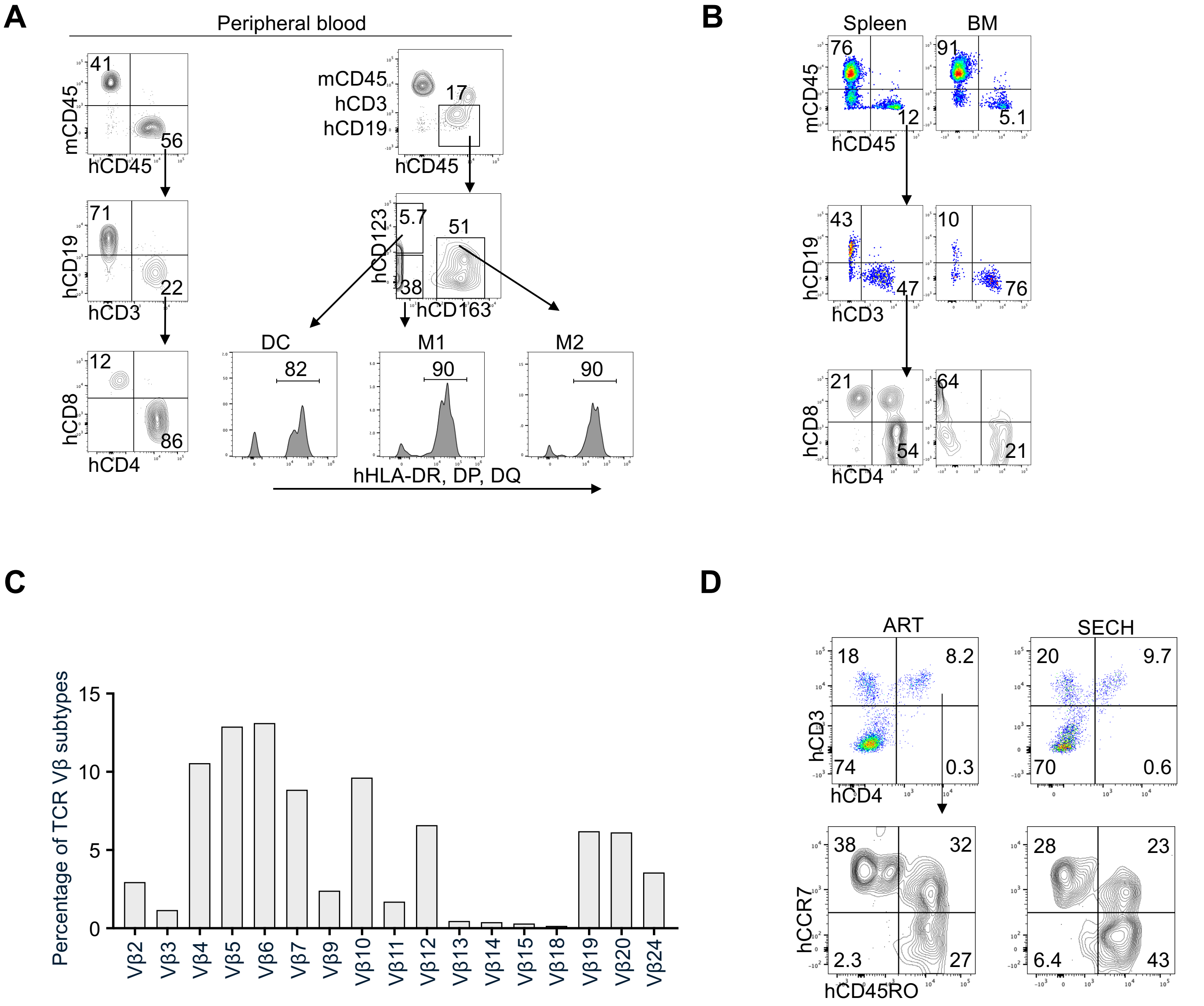

Supplementary Figure 1. Analyses of Hu-HSC mice used for HIV infection and cure studies. (A) Analysis of human immune cells in the peripheral blood. (B) Analysis of human lymphocytes cells in the spleen and bone marrow (BM). (C) T cell receptor Vβ usage by scRNA-seq in human CD4^{+} T cells sorted from Hu-HSC mice. (D) Spleen cells from Hu-HSC mice treated with SECH or ART were analyzed by flow cytometry for CCR7^{+}CCD45RO^{-} naïve T cells, CCR7^{+}CCD45RO^{+} central memory T cells, CCR7^{-}CCD45RO^{+} effector memory T cells and CCR7^{-}CCD45RO^{-} activated effector T cells.

A

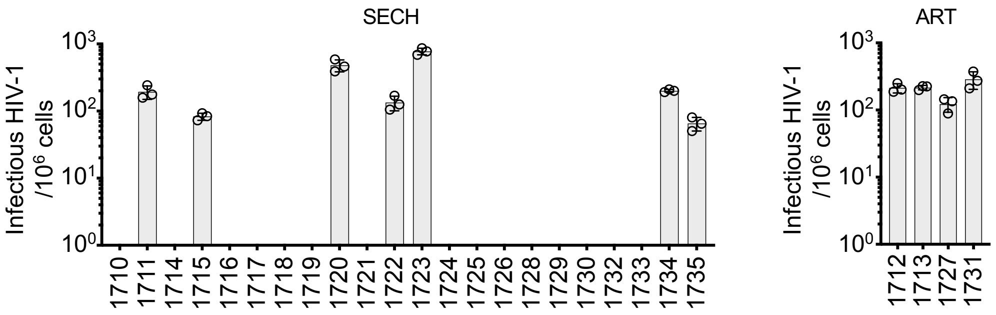

B

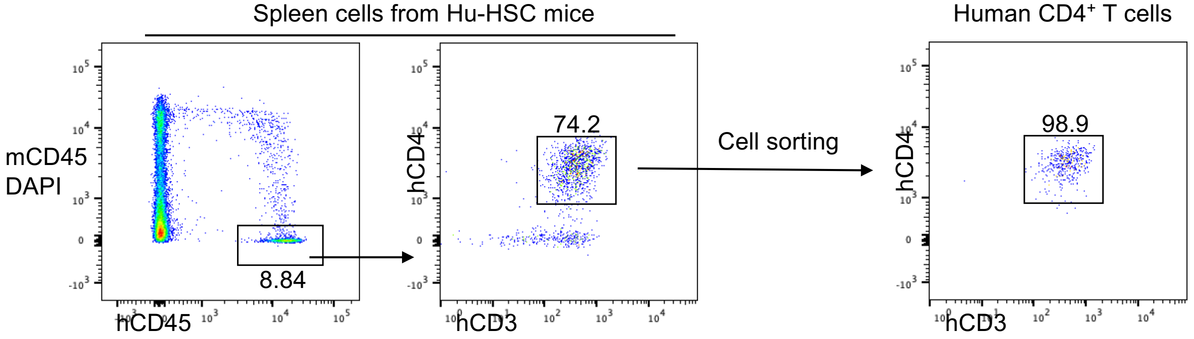

C

Clusters		Cluster name	Annotation of clusters
1	■	Tscm	CCR7+, CD27+, SELL+, CD95+, CD122+
2	■	Tem	CCR6+, IL15R+, KLRB1+, SELL-
3	■	Tem precursor	IL7R+, CD44-, KLRB1-, CD11a-, SELL-, TBX21-
4	■	Tem	IL7R+, CD44-, KLRB1+, CD11a+, TBX21+
5	■	Actvated T	CD69+, CD44+, CCR7-, PDCD1+
6	■	Tfh	BCL6+, PDCD1+, NMB+, IL21+
7	■	Tcm	CCR7+, SELL+, CD44+, TCF7+
8	■	Naïve T	CCR7+, CD27+, SELL+, FOXP1-, CD44-
9	■	Treg	FOXP3
10	■	Cytotoxic	GZMK+, GZMA+, CXCR3+, GZMH+
11	■	Exhausted T cell	CD3E+, PDCD1+, TIGIT+, LAG3+
12	■	Proliferating T	MKI67+
13	■	NKT	CXCR3+, IRF7+, NKG7+, CD56+

Supplementary Figure 2. Purification of human $CD4^+$ T cells from Hu-HSC mice for single cell RNA sequencing. (A) Quantification of infectious HIV-1 in the spleen of mice treated by SECH or ART by TZA assays. (B) The strategy for purification of human $CD4^+$ T cells from Hu-HSC mice. (C) The annotation of clusters in UMAP.

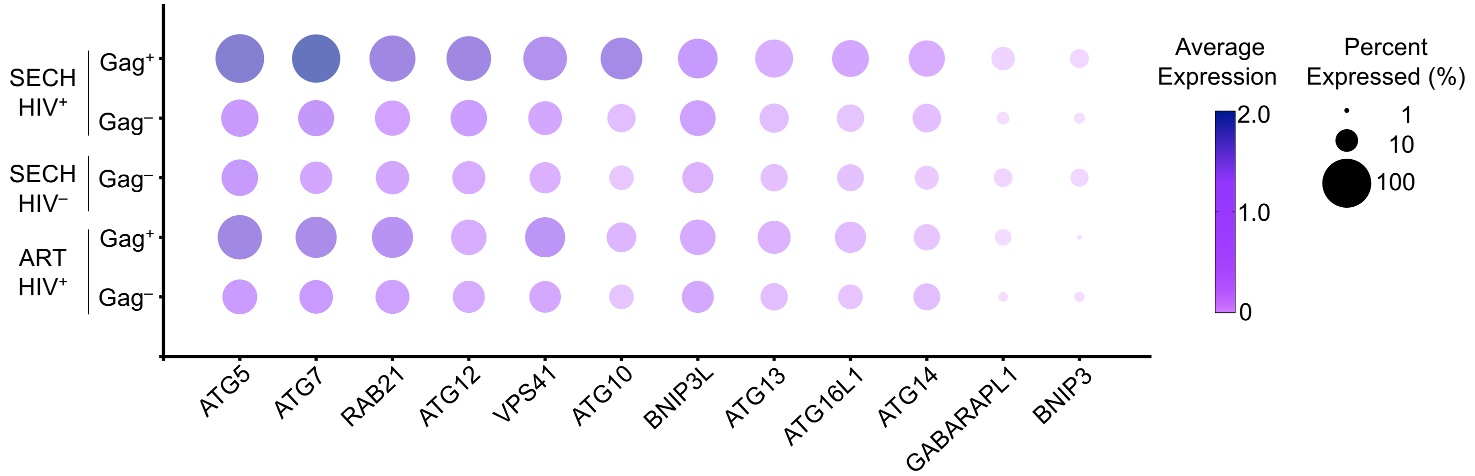

Supplementary Figure 3. Autophagy gene expression in Gag⁺ T cells from SECH-treated Hu-HSC mice. Bubble plot depicts the average level of normalized expression of autophagy genes in Gag⁺ or Gag⁻ CD4⁺ T cells in humanized mice with failed HIV clearance by SECH (SECH-HIV⁺), Gag⁻ CD4⁺ cells with successful HIV clearance (SECH-HIV⁻), and Gag⁺ or Gag⁻ CD4⁺ T cells from mice treated by ART. The color of bubble represents the average level of normalized expression of the genes of indicated. The size of bubble represents the percentage of cells expressing the genes of indicated.

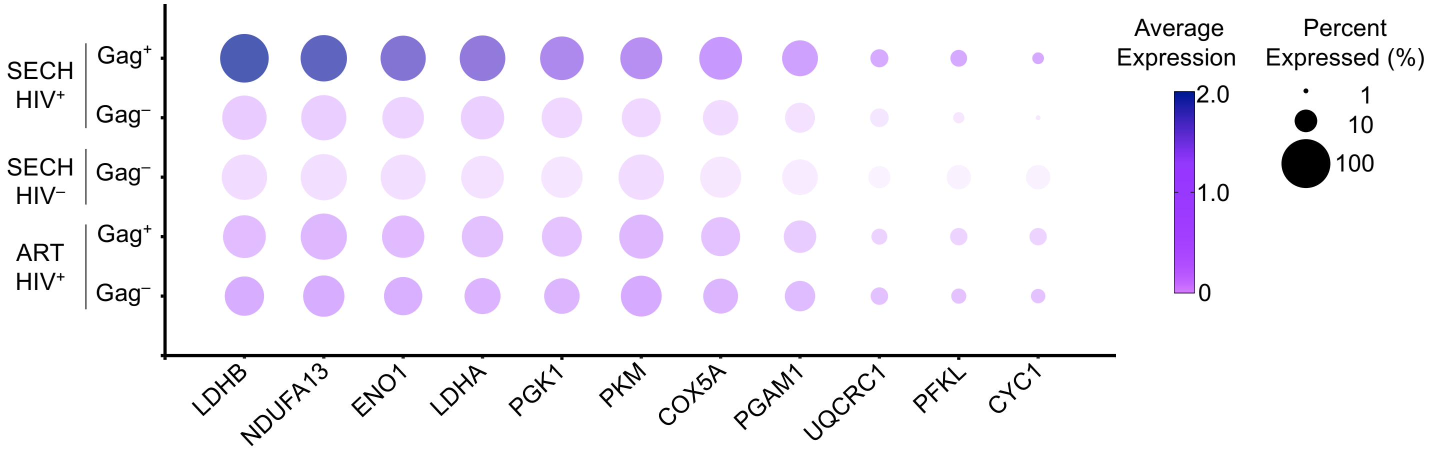

Supplementary Figure 4. Glycolysis gene expression in Gag^+ T cells from SECH-treated Hu-HSC mice. The level of normalized expression of glycolytic genes in Gag^+ or Gag^- $CD4^+$ T cells in humanized mice with failed HIV clearance by SECH (SECH-HIV^+), Gag^- $CD4^+$ cells with successful HIV clearance (SECH-HIV^-), and Gag^+ or Gag^- $CD4^+$ T cells from mice treated by ART. The color of bubble plot represents the levels of normalized expression of the genes of indicated. The size of bubble represents the percentage of cells expressing the genes of indicated.

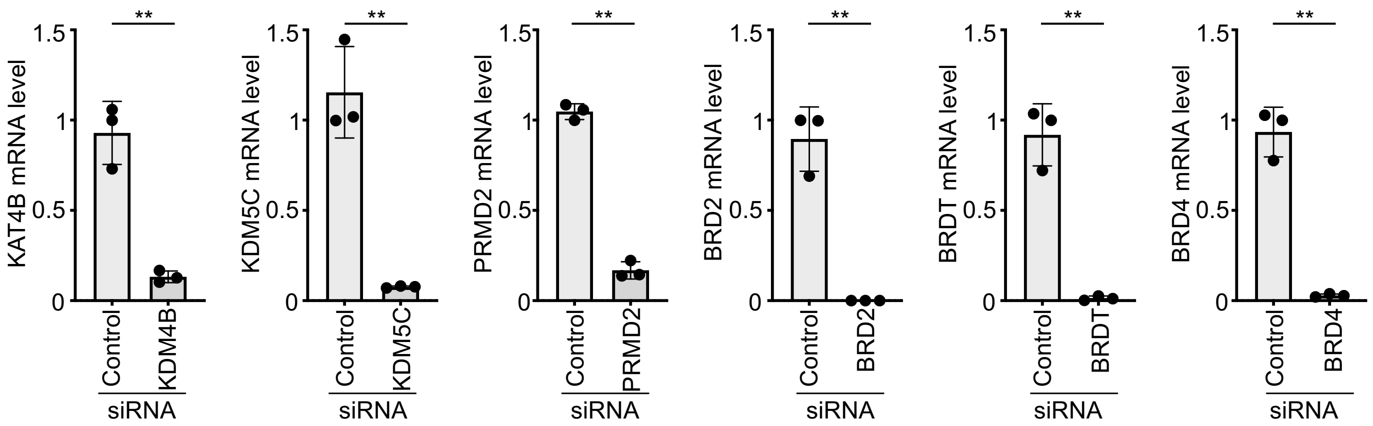

Supplementary Figure 5. Gene silencing in T cell. T cells were transfected with siRNAs targeting individual genes or control siRNA. The cells were used for RNA extraction and RT-PCR analysis to determine the expression of targeted genes. $^{**}P<0.01$

A

ART-experienced PLWH

Patient	Race	Sex	Age	CD4 Count	CD4 %	HIV RNA Levels (copies/mL)
PT1	B	M	34	122	9	–
PT2	B	M	67	88	11	–
PT3	C	F	60	829	32	–
PT4	C	M	67	754	37	ND
PT5	NR	M	58	931	22	–

B, black

C, caucasian

NR, information not released

ND, not detectable

–, not tested at the time of blood collection

B

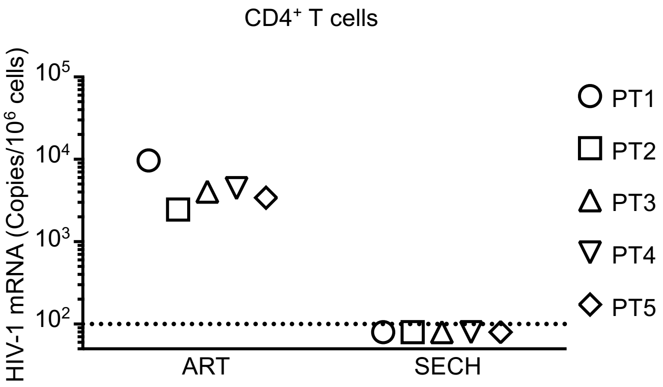

Supplementary Figure 6. PBMCs from ART-experienced PLWH used for SECH treatments. (A) The information of ART-experienced PLWH used in this study. (B) $CD3^{+}CD4^{+}$ T cells were sorted from patient PBMCs for treatments with SECH regimens or ART control. The cells were used for determination of HIV-1 mRNA by RT-PCR.
